# Plasmids of the urinary microbiota

**DOI:** 10.1099/acmi.0.000429

**Published:** 2022-11-30

**Authors:** Genevieve Johnson, Seanna Bataclan, Minerva So, Swarnali Banerjee, Alan J. Wolfe, Catherine Putonti

**Affiliations:** ^1^​ Bioinformatics Program, Loyola University Chicago, Chicago, IL, USA; ^2^​ Biology Program, Division of Natural Sciences, University of Guam, Mangilao, GU, USA; ^3^​ School of Life Sciences, Arizona State University, Tempe, AZ, USA; ^4^​ Department of Mathematics and Statistics, Loyola University Chicago, Chicago, IL, USA; ^5^​ Department of Microbiology and Immunology, Loyola University Chicago, Maywood, IL, USA; ^6^​ Department of Biology, Loyola University Chicago, Chicago, IL, USA

**Keywords:** microbiome, plasmid, plasmidome, urinary tract, urobiome

## Abstract

Studies of the last decade have identified a phylogenetically diverse community of bacteria within the urinary tract of individuals with and without urinary symptoms. Mobile genetic elements (MGEs), including plasmids and phages, within this niche have only recently begun to be explored. These MGEs can expand metabolic capacity and increase virulence, as well as confer antibiotic resistance. As such, they have the potential to contribute to urinary symptoms. While plasmids for some of the bacterial taxa found within the urinary microbiota (urobiome) have been well characterized, many urinary species are under-studied with few genomes sequenced to date. Using a two-pronged bioinformatic approach, we have conducted a comprehensive investigation of the plasmid content of urinary isolates representative of 102 species. The bioinformatic tools plasmidSPAdes and Recycler were used in tandem to identify plasmid sequences from raw short-read sequence data followed by manual curation. In total, we identified 603 high-confidence plasmid sequences in 20 different genera of the urobiome. In total, 70 % of these high-confidence plasmids exhibit sequence similarity to plasmid sequences from the gut. This observation is primarily driven by plasmids from *E. coli *, which is found in both anatomical niches. To confirm our bioinformatic predictions, long-read sequencing was performed for 23 of the *E. coli* isolates in addition to two *E. coli* strains that were sequenced as part of a prior study. Overall, 66.95 % of these predictions were confirmed highlighting the strengths and weaknesses of current bioinformatic tools. Future studies of the urobiome, especially concerning under-studied species in the urobiome, should employ long-read sequencing to expand the catalogue of plasmids for this niche.

## Full-Text

## Data Summary

Whole-genome sequencing using the Nanopore long-read technology was conducted for 23 urinary *E. coli* isolates. The raw reads have been deposited in NCBI’s SRA database. They are deposited with prior Nanopore long-read sequencing of two urinary *E. coli* isolates also examined as part of this study. This data can be found under the following accession numbers: SRR17649134 through SRR17649156. Accession numbers for additional data used in this study are included in, available in the online version of this article.

**Impact Statement**

Plasmids have the potential to significantly impact the ecology and evolution of microbial communities. With respect to human health, plasmid-mediated antibiotic resistance is a significant concern. Recently, we investigated the plasmid population of bacteria within the urinary tract, focusing on representatives of clinically relevant species. Many species of the urinary microbiota are under-studied with few genomes sequenced to date. Consequently, plasmids have yet to be explored. Here we present the first catalogue of plasmids for the urinary microbiota using publicly available short-read sequencing data. We identified these plasmid sequences using complementary bioinformatic tools coupled with manual curation. Our investigation exposes limitations of individual bioinformatic approaches and challenges of examining under-studied taxa. We find that long-read sequencing is a reliable means of validating our plasmid predictions. This study provides the first investigation of plasmids in 102 species commonly found in the human urinary tract. It also presents a reference for bioinformatic methods for the identification of plasmids in under-studied species.

## Introduction

As microbiome research progresses, we have transitioned from identification of the taxon present to investigation of the functionality encoded. This transition broadens our perspective from the chromosomal to also include the mobile genetic elements (MGEs) that persist within a microbial community, the mobilome. The mobilome consists of phages and plasmids that can confer increased fitness to their bacterial host cell, including expanding metabolic capacity, antibiotic resistance and virulence (see reviews [1–3]). With respect to human health, plasmid-mediated antibiotic resistance remains a significant concern and plasmids can drive niche specificity for a species [2, 4].

Recently, we investigated the plasmid population of bacteria within the urinary tract, focusing on representatives of 11 clinically relevant urinary species [5]. Prior research has found that plasmids can promote pathogenicity in the urinary tract, e.g. *Escherichia coli* urinary tract infections (UTIs) [6–8], *Klebsiella pneumoniae* UTIs [7] and *Acinetobacter baumannii* catheter-associated urinary tract infections [9]. Our prior bioinformatic analysis of 11 species led to the identification of plasmid sequences in strains of *E. coli *, *Enterococcus faecalis *, *K. pneumoniae *, *Staphylococcus epidermidis* and *Streptococcus anginosus* [5].

Numerous challenges limit the identification and characterization of plasmids in the human microbiome. First, plasmid databases are not comprehensive and are biassed towards well-studied organisms [10]. Thus, methods for plasmid identification that rely on database searches are inherently limited and thwart discovery of new plasmids. Second, plasmid sequences that contain repetitive elements or are present within multiple genomes (in the case of metagenomic sequencing) are well-known issues for de Bruijn assemblers of short-read sequences [11]. As a result, bioinformatic tool developers have taken different approaches for plasmid discovery. Recent comparisons of these tools found limitations for all contemporary tools [12, 13].

Herein, we present an expanded analysis of the plasmidome of the urinary microbiota (urobiome), considering 102 different species that are representative of the phylogenetic diversity of the urobiome. In contrast to our prior work [5], we employed two different tools for plasmid detection: plasmidSPAdes [14] and Recycler [15]. While both tools utilize de Bruijn graphs for predicting plasmid sequences, plasmidSPAdes is tuned to identify contigs with sequencing coverages deviating from the average (e.g. high copy number plasmids), and Recycler is designed to identify circular contigs. Through our investigation of the plasmidome of the urobiome, we identified new plasmids, broad host-range plasmids and niche-specific plasmids. Complementing our two-pronged bioinformatic analysis, we conducted long-read sequencing to validate some of the plasmids identified.

## Methods

### Data retrieval

All SRA files for the female urinary microbiome project BioProject Accession Number PRJNA316969 were retrieved using SRA Toolkit’s prefetch function (https://github.com/ncbi/sra-tools; v. 2.11.0). Only strains that were collected from urine samples (*n*=397) were processed further . Isolation source was determined from the corresponding BioSample files; 392 were from samples collected via transurethral catheterization (TUC). Isolates from TUC samples are from the urinary tract rather than contaminants introduced through voiding (see review [16]). The SRA format files were converted to fastq format using SRA Toolkit’s fastq-dump function. Raw reads were trimmed using bbduk (v. 38.90), part of the BBtools suite (https://sourceforge.net/projects/bbmap/) with the following parameters minlength=30 qtrim=rl maq=20 maxns=0 trimq=20.

### Plasmid prediction

The trimmed reads were used as input to plasmidSPAdes (v. 3.15.2) [14] and Recycler (downloaded 27 June 2021) [15]. Recycler takes as input the results of SPAdes and BWA. We used SPAdes v. 3.15.2 and BWA v. 0.7.17 to process the trimmed reads and produce input for Recycler. SPAdes was run with the ‘--only-assembler’ option for k values of 55, 77, 99 and 127. BWA was used to generate the BAM files required for Recycler (see commands here: https://github.com/Shamir-Lab/Recycler). Plasmids were manually curated as follows. Predicted plasmid sequences were uploaded to NCBI’s blast website and queried (using the megablast algorithm) against the nr/nt database. Results were examined. If the top hit(s) were to plasmid sequence records with a query coverage and sequence identity >70 %, the predicted plasmid sequence was considered to be a (or representative of a) plasmid sequence. Predicted plasmid sequences also were queried against the Plasmid Database PLSDB (v. 2020_11_19v0.4.1–9) [17] locally using blastn (v. 2.9.0). If a predicted plasmid sequence only produced hits to chromosomal sequences in nr/nt and there were no hits to PLSDB records, the predicted plasmid sequence was considered to be a false-positive result. In parallel, predicted plasmid sequences were examined using CGE’s (the Center for Genomic Epidemiology) PlasmidFinder (v. 2.0.1) using the default parameters [18]. If either the blasts (nr/nt and PLSDB) or PlasmidFinder queries returned results indicative of a plasmid sequence, the predicted plasmid sequence was considered further; we refer to these as high-confidence plasmid sequences.

To ascertain if a plasmid sequence was predicted by both tools, reciprocal blast was performed locally using the blastn algorithm. If plasmid sequences shared >50 % query coverage, they were considered matches. One Recycler prediction was equivalent to the concatenation of two plasmidSPAdes predictions. All other ‘matches’ between the plasmidSPades and Recycler predictions were unique.

### Plasmid sequence comparisons

Three types of comparisons were conducted for the high-confidence plasmid sequences. First, high-confidence plasmid sequences were examined for the presence of virulence factors and antibiotic resistance genes. The databases from ResFinder [19] and VFDB [20] were retrieved and blast queries were run locally using blastn. Second, high-confidence plasmid sequences were compared to each other using Mash (v 2.2) [21]. An adjacency matrix and clusters of homologous plasmid sequences were determined using a Python script (available at https://github.com/putonti/urobiome_plasmids). A Mash distance of <0.01 was used as a threshold for homology. Lastly, high-confidence plasmid sequences were compared to plasmid sequences from the gut from the mMGE database [22]. Local blastn queries were performed against the mMGE database; a threshold of 70 % query coverage and 70 % identity were used to determine matches. All statistical analyses conducted in this study were performed using R (v4.1.2).

### Nanopore sequencing of urinary *E. coli* isolates

From the genomes examined, 23 strains of *E. coli* were selected for long-read sequencing. These strains include representatives of diverse computationally predicted plasmids: UMB0934, UMB0939, UMB1160, UMB1161, UMB1193, UMB1221, UMB1223, UMB1225, UMB1229, UMB1335, UMB1337, UMB1346, UMB1360, UMB1362, UMB3641, UMB5924, UMB5978, UMB6471, UMB6653, UMB6655, UMB6721, UMB7431 and UMB9182. These strains were isolated as part of prior IRB-approved studies. These strains, stored at −80 °C, were streaked onto LB plates (1.7 % agarose) and grown overnight at 37 °C. A single colony was pulled from each plate and grown in 1 ml of LB media overnight at 37 °C. The plasmid DNA of the culture was extracted using the QIAmp DNA Mini Kit with a modified protocol. An initial centrifugation at 5000 ***g*** for 10 min to pellet the bacteria and resuspension in 180 uL of Buffer ATL was added to the protocol. The sample was not vortexed when specified in the protocol; rather samples were mixed by shaking by hand.

The extracted DNA was sent to Columbia University (New York, NY, USA) for Nanopore DNA sequencing. Sequencing libraries were prepared using the Rapid Barcoding 96 kit (Oxford Nanopore, SQK-RBK110.96) according to the manufacturer’s instructions. Libraries were sequenced on an Oxford Nanopore GridION using an R9.4.2 flow cell. High-accuracy basecalling and demultiplexing was performed using MinKNOW v21.05.20. Barcode sequences were removed using Porechop v0.2.3, and Mothur v1.25.0 was used to remove short-read fragments (min. length 1000 bp) and filter homopolymeric reads (max. homopolymeric length 20 bp). Additionally, two isolates, UMB1180 (SRA Accession No. SRR16938658) and UMB1284 (SRA Accession No. SRR16938656), were sequenced by the same methods as part of a prior study [5]. Thus, 25 of our strains examined via plasmidSPAdes and Recycler were sequenced via long-read Nanopore sequencing.

We next conducted hybrid assemblies, using the publicly available Illumina raw reads () and the Nanopore sequencing data produced here (see Data Summary). The hybrid assembly was performed using Unicycler v.0.4.9 with default parameters [23]. Plasmid sequences were identified with a local blast search against the PLSDB plasmid database. The sequences were also compared to *E. coli* str. K-12 MG1655 (GenBank Accession No. NC_000913) to remove misidentified *E. coli* chromosome sequences using a local blast search. Sequences that had >90 % identity and >50 % query coverage to plasmid sequences in PLSDB [17] were retained as probable plasmid sequences. Sequences that had >90 % identity and >50 % query coverage to *E. coli* str. K-12 were probable *E. coli* chromosome sequences. Sequences that matched to both plasmids and *E. coli* K-12 were manually examined and queried against the NCBI nr/nt database. Select plasmid sequences were annotated using RAST with the RASTtk option [24, 25].

Final identified plasmid sequences from the 25 *E. coli* isolates were compared to the computationally identified urinary plasmid sequences using a local blast. Resulting matches with greater than 90 % nucleotide identity and greater than 90 % coverage were considered to be shared plasmids between the *E. coli* sequencing data and the predicted high-confidence plasmid sequences from the Illumina data.

Assemblies were also conducted using just the Nanopore sequencing reads using Unicycler v.0.4.9 with default parameters [23]. The assembled contigs were compared to the *E. coli* plasmid sequences from the hybrid assembly via local blast. Hits with a query coverage of >90 % (regardless if they matched in one alignment or more than one [to accommodate differences in rotation]) were considered to be shared plasmid sequences.

## Results

### Bioinformatic prediction of plasmid sequences

Raw fastq format data from 397 isolates from the female urinary microbiota were examined for the presence of plasmids. In total, 98.7 % of these isolates were obtained via transurethral catherization and thus are representatives of microbes of the bladder urine. This data set includes 54 different genera and 102 different species (Table S1). Two plasmid prediction tools were used: plasmidSPAdes and Recycler. Altogether, 4959 plasmid sequences were identified by plasmidSPAdes and 634 plasmid sequences were identified by Recycler. Overall, 336 of these plasmids were predicted by both tools. We next manually curated these predicted plasmid sequences. As described in Methods, each predicted plasmid sequence was queried against the nr/nt database as well as examined using the CGE PlasmidFinder tool online. This process of manual curation resulted in 603 high-confidence plasmid sequences (Table S1). Table 1 lists the genera containing high-confidence plasmid sequences. Most (71.64%) of these sequences were identified in *E. coli* strains.

**Table 1.** Summary statistics of genera containing high-confidence plasmids

Genus	No. of strains examined	No. of strains containing high-confidence plasmid sequence(s)	No. of high-confidence plasmid sequences
* Acinetobacter *	1	1	9
* Actinomyces *	10	1	1
* Aerococcus *	39	1	1
* Alloscardovia *	2	1	1
* Bacillus *	3	1	1
* Enterococcus *	5	2	5
* Escherichia *	77	58	432
* Gleimia *	2	1	1
* Gordonia *	1	1	2
* Klebsiella *	6	4	63
* Lactobacillus *	53	14	20
* Limosilactobacillus *	2	2	11
* Micrococcus *	5	1	2
* Moraxella *	1	1	4
* Neisseria *	3	1	1
* Nosocomiicoccus *	2	1	2
* Prevotella *	2	1	1
* Pseudomonas *	13	1	5
* Staphylococcus *	16	9	30
* Streptococcus *	61	10	11

Of these high-confidence plasmid sequences, 406 were predicted only by plasmidSPAdes (average length=14.0 kbp), 105 only by Recycler (average length=7.4 kbp), and 92 by both plasmidSPAdes and Recycler (average length=13.6 kbp). Through the process of manually curating all plasmid predictions, three frequent observations were made with regards to the plasmidSPAdes plasmid sequences. First, plasmidSPAdes predicted sequences that included ribosomal RNA operons. This is expected, as the plasmidSPAdes algorithm identifies putative plasmid sequences with a significantly greater coverage than the average; most strains have multiple copies of this operon, which would result in greater sequencing of this region. Second, plasmidSPAdes results included phage sequences. This suggests active replication of the phage within the sequenced isolate. Third, plasmidSPAdes predictions often included multiple partial plasmid sequences. Through blast analysis, multiple plasmidSPAdes predictions for the same strain show sequence similarity to the same GenBank record. This suggests that the complete plasmid was not assembled yet plasmidSPAdes could identify the sequence. These partial plasmid assemblies were not detected by Recycler, as they could not be assembled into a circular contig.

### Characterization of the urobiome plasmids

High-confidence plasmid sequences were examined for the presence of antibiotic resistance genes and virulence factors. In total, 134 antibiotic resistance genes, from 76 different high-confidence plasmid sequences were identified. Species encoding these antibiotic resistance genes include *E. faecalis* (one gene), *E. coli* (116 genes), *S. aureus* (one gene), *S. epidermidis* (15 genes) and *S. anginosus* (one gene). These genes include predicted resistances to aminoglycosides (*n*=38), the folate pathway (*n*=32), beta-lactams (*n*=21), and tetracyclines (*n*=19). In total, 112 virulence factor genes were identified, from 54 different high-confidence plasmid sequences from 35 different strains. Two of these strains were *E. faecalis *, while the remaining plasmids encoding virulence factor genes were harboured by *E. coli* strains. Symptom status is available for all of the *E. coli* strains examined here, which include isolates from women with UTI symptoms, with overactive bladder symptoms (OAB), and without lower urinary tract symptoms (no LUTS). No difference was observed in the prevalence of antibiotic resistance genes (*P*-value=0.5382) or virulence factor genes (*P*-value=0.3802) between isolates from either symptom class or the no LUTS strains. Table S2 lists the genes of interest identified.

The high-confidence plasmid sequences were compared to plasmids sequenced from either gut isolates or from gut shotgun metagenomic studies. Of the 603 sequences, 416 exhibited significant sequence similarity to a gut plasmid (>70 % of the urinary plasmid sequence aligned to the gut plasmid sequence and >70 % of the gut plasmid sequence aligned to the urinary plasmid sequence). The majority (83.41%) of these similar plasmids were from the urinary *E. coli* strains. Altogether, 39 plasmids from urinary *K. pneumoniae* strains also were similar to the gut plasmids. Table S3 lists the other species with a high-confidence plasmid similar to a plasmid sequence from the gut.

### Diversity of urobiome plasmids

High-confidence plasmid sequences were compared to each other using the alignment-free method Mash (see Methods). There are 386 clusters of urobiome plasmids. Overall, 293 of these clusters include just a single plasmid indicating that the plasmid was unique to the strain. The largest cluster includes 19 plasmid sequences, found in 19 different urinary *E. coli* strains. The cluster includes sequences that vary in length, between 10.1 and 17.8 kbp, and when aligned has an average sequence identity of 84 %. The 10.1 kbp sequence is conserved amongst all of the sequences and shows identity to larger *E. coli* plasmid sequences in the nr/nt database. Most (94.62%) of the clusters with one or more plasmids include plasmids found within the same species. Five of these clusters include plasmid sequences from different species (Table 2). As the table denotes, four of the five clusters include sequences only identified by Recycler; the other cluster includes sequences only identified by plasmidSPAdes.

**Table 2.** Broad host-range plasmid sequences. Sequences belonging to the same cluster that are found in strains of different species

Cluster size	Species (no. plasmid sequences)	Length (bp)	Average pairwise identity
5*	* Escherichia coli* (4) * Nosocomiicoccus ampullae* (1)	4082–4091	99.9%
3	* Staphylococcus aureus* (1) * Staphylococcus epidermidis* (2)	4396–4737	89.9%
9*	* Escherichia coli* (8) * Nosocomiicoccus ampullae* (1)	2088–2113	99.4%
3*	* Escherichia coli* (2) * Klebsiella pneumoniae* (1)	1546	100.0%
2*	* Alloscardovia omnicolens* (1) * Lactobacillus gasseri* (1)	4807	100.0%

*Plasmids identified by Recycler only.

### Experimental confirmation of plasmid sequences

In total, 23 *E. coli* strains included in our bioinformatic analysis were resequenced using the long-read Nanopore technology, which enables whole plasmid sequencing. These strains were selected because they were predicted from the Illumina data to include high-confidence plasmids belonging to different clusters. Previously we had sequenced two other *E. coli* strains (see Methods). Thus, we produced hybrid assemblies of both short- and long-read data for 25 strains, looking for evidence of plasmids. All 25 contained at least one putative plasmid sequence. Manual curation of hybrid assemblies, using both the long and short reads, identified 118 putative plasmid sequences, ranging in size from 1240 bp to 154 370 bp (Table S4). The putative plasmid sequences share significant sequence similarity to characterized plasmids from not only *E. coli *, but also other Enterobacteriaceae (* Citrobacter *, *Shigella *, *Klebsiella *, *Salmonella* and *Enterobacter *) and *Serratia* (Table S4). Then, 36 of the 118 putative plasmid sequences from the hybrid assemblies were assembled from the long reads only (Table S5).

**Fig. 1.**
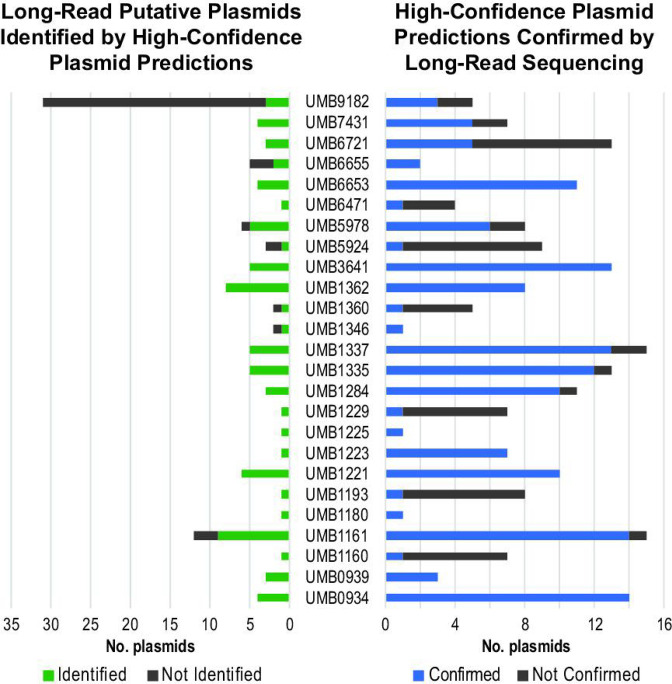
Comparison of the predicted plasmid sequences by long-read and short-read sequencing.

**Fig. 2.**
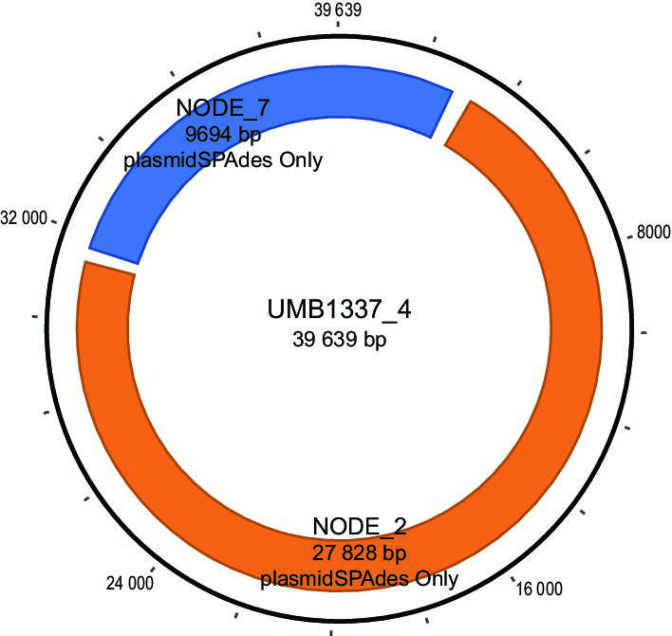
Alignment of plasmidSPAdes predicted high-confidence plasmid sequences (orange and blue arcs) to one of the plasmid sequences identified by long-read sequencing of *E. coli* UMB1337.

In total, 79 of the 118 putative plasmid sequences were identified by plasmidSPAdes and/or Recycler during the bioinformatic analysis of the Illumina-only assemblies (Fig. 1; Table S6). Both small and large plasmid sequences from the hybrid assemblies were identified by plasmidSPAdes and/or Recycler (average length of identified plasmids: 26 350 bp, minimum: 1240 bp, maximum: 154 370 bp; average length of unidentified plasmids: 10 527 bp, minimum: 2028 bp, maximum: 110 763 bp). Altogether, 57 of the hybrid assembled plasmids were ‘exact’ matches to high-confidence sequences from our bioinformatic analysis; in other words, the bioinformatic predicted plasmid sequence was nearly identical (99.12–100 % nucleotide sequence identity) to the assembled putative plasmid sequence. This included 14 high-confidence sequences predicted by both plasmidSPAdes and Recycler (average length=9.7 kbp), 17 predicted by plasmidSPAdes only (average length=14.0 kbp), and 26 predicted by Recycler only (average length=3.9 kbp). One high-confidence plasmid sequence was identified by the hybrid assembly as two individual putative plasmid sequences. Fragmented predictions – hybrid assembled plasmids that are represented by multiple high-confidence predicted plasmid sequences – primarily consist of sequences predicted by plasmidSPAdes only (*n*=76), with nine predicted by Recycler only, and two predicted by both tools (Table S6). Fig. 2 is an example of a plasmid assembled from the short- and long-read sequences that was predicted by plasmidSPAdes (only) as two different contigs. While one of these plasmidSPAdes contigs (NODE_7) contains the replication initiation protein RepE, the other (NODE_2) contains plasmid conjugative proteins, e.g. TraD, TraG (Table S7).

## Discussion

This study provides a catalogue of high-confidence plasmid sequences for bacteria of the urobiome. While a few of the taxa from the urobiome have been well-characterized with regards to their plasmids, e.g. *E. coli *, the majority have not. Among the 102 species examined, 432 of the high-confidence plasmids identified here were from *E. coli* strains. Furthermore, 51 of the 77 *E. coli* strains contained at least one high-confidence plasmid sequence. The two bioinformatic tools tested here, plasmidSPAdes and Recycler, identified 4959 and 634 plasmid sequences, respectively. Through manual curation, however, this number was greatly reduced. plasmidSPAdes predictions had a higher false-positive rate; of the 4959 predictions, only 356 of the plasmid sequences predicted only by plasmidSPAdes were identified as high-confidence plasmid sequences (Table S1). Prior benchmarking studies of plasmid prediction tools have similarly noted the low precision of plasmidSPAdes [12, 13]. In contrast, 197 of the 634 predictions by Recycler were determined to be high-confidence plasmid sequences (Table S1). While the precision of plasmidSPAdes (7%) was lower than that previously reported for the tool, the precision of Recycler (30%) was on par with prior reports [12, 13].

The low precision observed for plasmidSPAdes may be the result of biases entered during the manual curation process. The manual curation process included querying predicted plasmid sequences against known plasmid databases and GenBank. However, these resources are heavily biassed in the taxa represented [26]. For some of the genera examined here no or very few plasmid sequences have been identified and deposited in publicly available databases. This absence may be due to the fact that the genus does not support plasmids or the genus has not been sufficiently sequenced to capture species or strains harbouring plasmids. For many of the urobiome species investigated here, very few genome sequences are publicly available. Moreover, if these species do support plasmids, their plasmids may not share any resemblance to sequences from well-studied taxa, and thus were excluded during our manual curation and have been overlooked by other studies. Long-read sequencing of multiple strains of understudied taxa of the urobiome will provide a means of exploring their plasmid content.

While here we have selected two bioinformatic tools for plasmid prediction other tools exist, including tools that search for marker genes, e.g. PlasmidFinder [18], *k*-mer usage profiles, e.g. cBar [27], or hybrid (de Bruijn graph and marker gene) approaches, e.g. SCAPP [28]. Both plasmidSPAdes and Recycler utilize de Bruijn graph metrics for identifying plasmids based on coverage and circular paths in the de Bruijn graph, respectively. These two tools are optimized for detecting high-copy number plasmids and circular plasmids. Our two-pronged approach improved our ability to identify plasmids than either tool alone. plasmidSPAdes identified fragmented plasmid sequences that were not identified by Recycler in the Illumina short-read sequence data.

Long-read sequencing of the 25 *E. coli* isolates and performing hybrid assemblies confirmed many (66.95%) of the high-confidence predictions (Fig. 1) and frequently confirmed that the fragments identified by plasmidSPAdes indeed comprised a single plasmid sequence (Fig. 2). It is important to note that the long-read sequencing conducted here used different DNA material than the original short-read sequencing. The selected isolates were cultured from freezer stocks, the same freezer stocks that produced the short-read sequencing material. Thus, plasmids could have been lost during the re-culturing (or could have been lost in the culturing of the isolate for short-read sequencing). Plasmids often confer a cost to the bacteria in the absence of selection [29]. Furthermore, a different DNA extraction protocol was used for short-read and long-read sequencing, which may have contributed to the plasmid content sequenced. These factors may be contributing to the difference observed between the plasmids identified from long-read only assemblies and hybrid assemblies (Table S5).

Similar and identical plasmids between urobiome isolates were found to be harboured by different genera (Table 2), suggesting that plasmid exchange may occur between different species in the urobiome. Plasmid exchange has been well-documented between *E. coli* and other Gram-negative species [30, 31]. Comparison of the urinary plasmid sequences to plasmids from the gut microbiome revealed significant overlap (68.99%). This is primarily driven by *E. coli* plasmids found in both niches. Prior studies have found that the gut can be a reservoir for *E. coli* associated with UTIs [32–36]. Several species of the bladder microbiota, however, are not commonly found in the gut [37]. While the bacterial taxa of the vaginal and urinary microbiota are more similar [37], there is limited data about plasmid sequences for the vaginal community. Future studies characterizing and sequencing isolates from the vaginal community will provide insight into the shared plasmid content of these interconnected microbiota.

Future long-read sequencing of urobiome isolates will expand our understanding of plasmids in the urobiome, including understudied and urobiome-specific taxa. Further examination of plasmid sequences within the urobiome will also provide insight into their contribution to symptoms. Most antibiotic resistance (86.57%) and virulence (91.07%) genes were found in plasmids of *E. coli* strains. There was no association between the symptom status of the individual from which the *E. coli* came and the presence of either antibiotic resistance or virulence genes was found. While horizontally acquired antibiotic resistance remains a concern and impediment for treatment [38], our analysis finds that plasmid-mediated resistance is primarily associated with *E. coli* in the urobiome (Table S2). Although plasmid-mediated antibiotic resistance is well documented in *K. pneumoniae* [39], the *K. pneumoniae* plasmids identified here did not harbour antibiotic genes. Further sequencing of urinary *K. pneumoniae* isolates is needed to ascertain if this is characteristic of urinary strains.

Characterizing the mobilome of the urinary tract is an essential next step in understanding the urobiome and its contribution to the presence or absence of urinary symptoms. While there are several bioinformatic tools for identifying plasmid sequences [14, 15, 18, 27, 28], their limitations have been well-noted [12, 13]. Here we have shown that a multi-tool approach coupled with manual curation performs well for well-characterized species, confirmed by long-read sequencing. However, long-read sequencing is vital for accurately cataloguing the urinary mobilome. Future studies of the gene content of these plasmids may elucidate a taxon’s ability to colonize the urinary tract.

## Supplementary Data

Supplementary material 1

Supplementary material 2

Supplementary material 3

Supplementary material 4

Supplementary material 5

Supplementary material 6

Supplementary material 7
